# Non-contact photoacoustic imaging with a silicon photonics-based Laser Doppler Vibrometer

**DOI:** 10.1038/s41598-024-74266-y

**Published:** 2024-10-03

**Authors:** Emiel Dieussaert, Roel Baets, Hilde Jans, Xavier Rottenberg, Yanlu Li

**Affiliations:** 1 Photonics Research Group, Ghent University-IMEC, Technologiepark-Zwijnaarde 126, 9052 Ghent, Belgium; 2 Center for Nano- and Biophotonics, Ghent University, Technologiepark-Zwijnaarde 126, 9052 Ghent, Belgium; 3 IMEC, Kapeldreef 75, 3001 Leuven, Belgium

**Keywords:** Photoacoustic imaging, Laser Doppler Vibrometer (LDV), Silicon Photonics, Remote detection, Integrated optics, Optical sensors, Photoacoustics

## Abstract

Photoacoustic imaging has emerged as a powerful, non-invasive modality for various biomedical applications. Conventional photoacoustic systems require contact-based ultrasound detection and expensive, bulky high-power lasers for the excitation. The use of contact-based detectors involves the risk of contamination, which is undesirable for most biomedical applications. While other non-contact detection methods can be bulky, in this paper, we demonstrate a proof-of-concept experiment for compact and contactless detection of photoacoustic signals on silicone samples embedded with ink-filled channels. A silicon photonics-based Laser Doppler Vibrometer (LDV) detects the acoustic waves excited by a compact pulsed laser diode. By scanning the LDV beam over the surface of the sample, 2D photoacoustic images were reconstructed of the sample.

## Introduction

Over the past decade, photoacoustic imaging (PAI) has gained significant attention in biomedical fields^1–4^, including cancer detection^4,5^, vascular imaging^5–7^, and functional brain imaging^8^among others. In this technique, the absorption of light by tissue chromophores causes local heating and expansion, which results in the emission of an acoustic wave. Detection of these acoustic waves allows the reconstruction of the absorption profile in the sample. The combination of light for excitation and sound for detection allows for going beyond existing optical techniques, like optical microscopy or optical coherence tomography (OCT)^9^, in terms of imaging depth.

In conventional PAI systems, ultrasound waves generated through the photoacoustic effect are detected using contact-based detectors, typically ultrasound transducers^4^. Due to the large impedance mismatch between the sample and air and the high absorption of ultrasound in air, these detectors require direct contact with the sample of interest, often necessitating the use of coupling media such as gel or water to ensure efficient acoustic coupling^10^.

While contact-based detectors such as piezoelectric and capacitive micromachined transducers^11,12^have proven to be effective for many applications, direct contact between the detector and a biomedical sample or patient, often facilitated by coupling gels, presents a risk of contamination^13^. Moreover, most detectors are opaque and thereby limit efficient excitation light delivery. While most research on ultrasonic transducers focuses on decreasing the size or increasing the sensitivity^14^, our approach aims to remotely detect the ultrasound signals.

Over the past decades, optical techniques have been used as a non-contact alternative detection method^15^. One such promising approach involves the use of laser Doppler vibrometry (LDV). LDV is a well-established non-contact optical technique for measuring surface vibrations, and it has recently shown promise for the remote detection of the photoacoustic waves generated in PAI^16,17^. This non-contact approach eliminates mechanical coupling artifacts, reduces the risk of sample damage, and enables the study of delicate biological samples.

Photoacoustic imaging often requires detection of the acoustic waves at various locations on the surface of the sample. Most LDV systems, however, are limited to only a few detection beams and therefore require scanning the surface of the sample, creating a complex and expensive system and compromising the imaging speed. A solution for this could be to create multi-beam LDVs. However, conventional LDVs are fiber- or free-space-based systems and use discrete components for each beam, which makes scaling the number of detection points bulky and expensive.

Over the past few decades, silicon photonics has steadily gained recognition as a reliable platform for integrated optics, enabling the miniaturization and large-scale integration of optical components. The development of on-chip LDV systems based on silicon photonics has the potential to overcome the drawbacks of current LDV systems, providing compact and relatively cheap multibeam LDVs^18,19^.

The primary objective of this work is to demonstrate the feasibility of using a silicon photonics-based LDV system as a detector for photoacoustic measurements and to assess the limitations of this approach. Additionally, we aim to employ a compact laser diode (LD) source as the photoacoustic excitation source, which will contribute to reducing the overall size and cost of the setup^20^.

## Methods

**Fig. 1:**
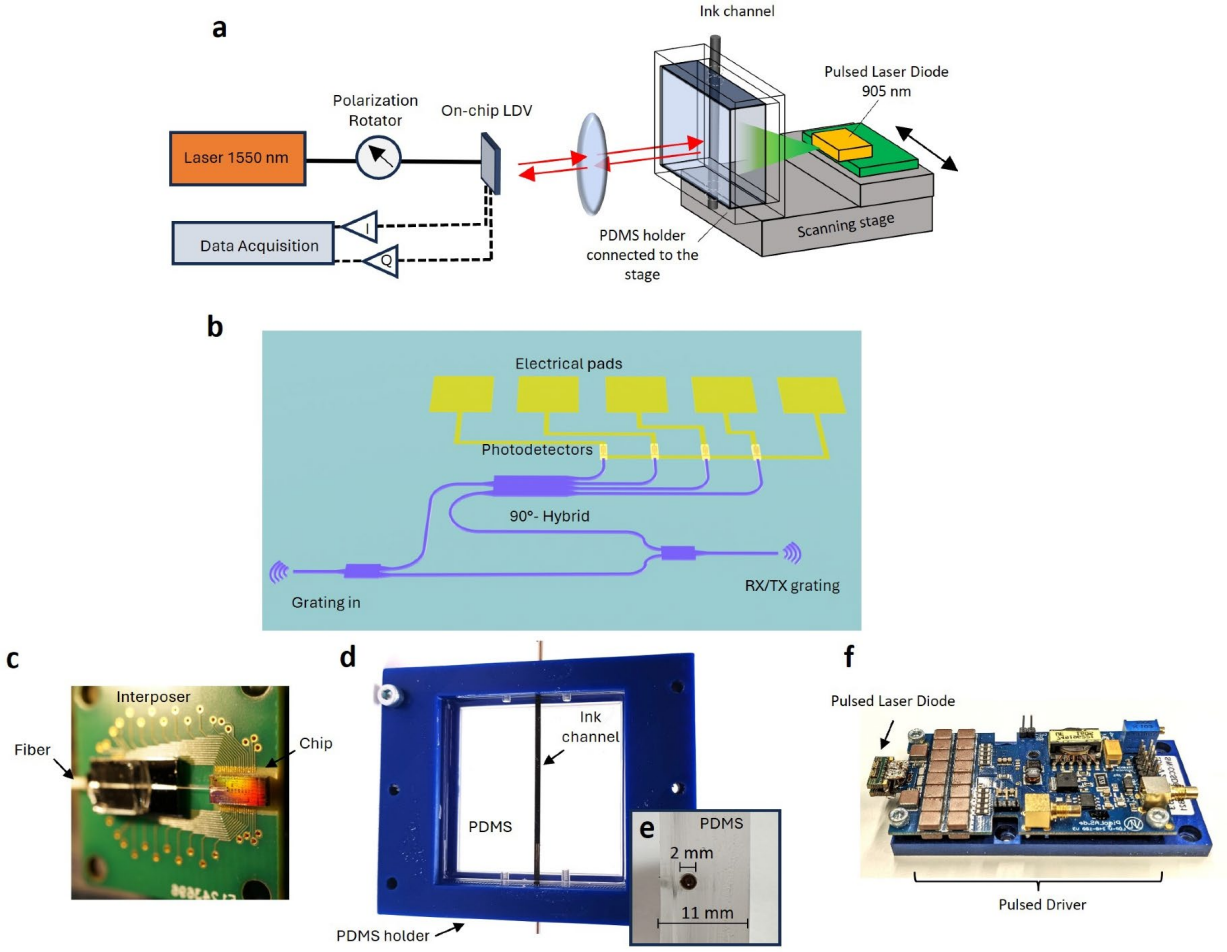
(**a**) A photoacoustic setup consisting of an on-chip LDV with a 1550 nm laser, connected to a data acquisition module. The probe beam is delivered by an optical lens system to the surface of the sample. On the right side of the sample, the pump laser diode fires optical pulses toward the silicone sample where the ink in the channel absorbs the light, hereby generating acoustic waves. (**b**) Schematic of an on-chip homodyne LDV where light is entering the chip through ’Grating in’ and after splitting into a reference and measurement arm is probing the target using the RX/TX grating. Reference and probe light are combined in a 90-degree optical hybrid and 5 electrical pads are used to read out 4 photodetectors. (**c**) Picture of the optical chip wire-bonded to an interposer PCB and an optical fiber glued to the input grating. (**d**) and (**e**) Pictures of the transparent silicone with an ink-filled embedded channel and its cross-section. (**f**) A picture of the 905 nm laser diode connected to the Picolas pulsed laser driver.

A photoacoustic system can be divided into three main elements: first, the excitation light source; second, the sample converting the excitation light into an acoustic signal; and lastly, an acoustic detection method. Figure 1a shows a schematic of the setup used in this paper. On the left side of the sample in Fig. 1a., there is the contactless detection system with the probe beam directed toward the sample, consisting of a laser source, the photonic integrated circuit (PIC), and the data acquisition module. On the right side of the sample, a 905 nm pulsed laser diode acts as the photoacoustic excitation source directed towards the silicone sample with an embedded ink channel that absorbs the excitation light and mimics the acoustic properties of a biological sample.

The photoacoustic signals generated from the ink channel travel through the silicone toward the sample’s surface, inducing small vibrations on the surface, which are detected by the chip-based LDV. To detect the small photoacoustic signals, averaging over multiple excitations was enabled using the triggered acquisition of LDV-detected signals synchronized with the firing of the pulsed diode.

As mentioned before, photoacoustic imaging requires the detection of the signals at multiple locations. Although photonic integration could allow for the dense integration of multi-beam LDVs, for this paper, a single-beam LDV was used while moving the sample and laser diode, both attached to the same scanning stage.

### Silicon photonics-based Laser Doppler Vibrometer

#### Device and working principle

A homodyne LDV is an optical interferometric device used to measure vibrations. It works by splitting a laser beam into a reference and measurement path. The measurement beam is directed at the vibrating surface, and when the reflected beam is combined with the reference beam on a photodetector, the resulting photocurrent depends on the relative phase of both beams. In this paper, we use a Silicon-On-Insulator platform to develop an integrated homodyne LDV. The high index contrast allows for the miniaturization of many optical components, fabricated using CMOS-compatible techniques. Here, we use IMEC’s ISIPP50G platform to integrate most of the optical components after the coupling of light^21^. A fiber-coupled distributed feedback laser (DFB) delivers 1550 nm-light to the PIC through a fiber glued to couple into the input grating coupler using a polarization rotator to ensure correct polarization into the chip. Figure 1b shows a schematic of the on-chip LDV which acts as a Mach-Zehnder interferometer. Once the light is coupled into the chip, the optical power is split into a measurement and reference path. The light in the measurement waveguide is directed towards a transmit/receive grating coupler (RX/TX grating). There, it is coupled out from the chip and focused on the target using a lens system. After reflection from the target, it is coupled back to the chip. Using an optical power meter at the position of the target, it was measured that around 0.5 mW is coupled from the chip to the target. This is within the eye-safety limits at this wavelength according to the ANSI standards^22^.

Back in the chip, the probe light is combined with the reference light using a 90-degree optical hybrid on the PIC^23^. The 90-degree optical hybrid is implemented as a 2-by-4 multimode interferometer (MMI). The field of the two input ports (one for the reference and one for the measurement light) excite multiple orders in the multimode region with each mode having a different propagation constant such that they interfere along the length of the MMI. Due to the relation between the propagation constants following the self-imaging principle, replicas of the input excitation are imaged. By properly placing the output waveguides, the light can be coupled into four outputs for which the phase relations between the reference and measurement field for each output port have a different 90-degree relative phase shift. We can express the optical field at the input of the MMI for the reference ($\textbf{R}$) and measurement field ($\mathbf {M(t)}$) using phasor notation:

1 $$\begin{aligned}&\textbf{R}=r \exp (i\theta _0) \end{aligned}$$

2 $$\begin{aligned}&\textbf{M}(t)=m \exp [i(\theta _1+\theta (t))] \end{aligned}$$

where $\theta _0$ and $\theta _1$ represent the static phase of the fields, while $\theta (t)$ contains the changing phase due to the path length change in the measurement arm. The outputs of the optical hybrids are combinations of the reference and measurement fields, which are converted into photocurrents by on-chip photodetectors. These combinations, expressed as $\textbf{M}(t)+ \textbf{R}$, $\textbf{M}(t)- \textbf{R}$, $\textbf{M}(t)+i \textbf{R}$, $\textbf{M}(t)-i \textbf{R}$, correspond to photocurrents $i_1(t),\;i_2(t),\;i_3(t),\;i_4(t)$ respectively. These currents can be combined into pairs to derive the in-phase (I) and out-of-phase (Q) components. Substituting $\theta '(t) = \theta (t)+\theta _1-\theta _0$, and considering the responsivity of the photodiodes as $\mu$, the combinations can be expressed as:

3 $$\begin{aligned} I(t)&= i_1(t) - i_2(t) = 2 \mu |rm| \cos (\theta '(t)) \end{aligned}$$

4 $$\begin{aligned} Q(t)&= i_3(t) - i_4(t) = 2 \mu |rm| \sin (\theta '(t)) \end{aligned}$$

The photodetector signals are transferred to a printed circuit board through 5 wirebonded electrical pads (1 ground and 4 signals) from the PIC to the PCB (Fig. 1b,c). Hereafter, differential amplification of these current signal pairs performs the combination according to Eq.3 and 4, resulting in the in-phase (I) and quadrature (Q) electrical signal. After amplification, the I and Q signals are recorded using a data acquisition card (Gage; both channels have a sampling rate of $65 \times 10^6$ samples per second). From these recorded signals, the relative phase difference $\theta '(t)$ between the reference and measurement beam can be demodulated using Eq. 5, and as such, relative movements of the target can be detected.

5 $$\begin{aligned} \theta '(t) = \arctan (\frac{Q(t)}{I(t)}) \end{aligned}$$

**Fig. 2:**
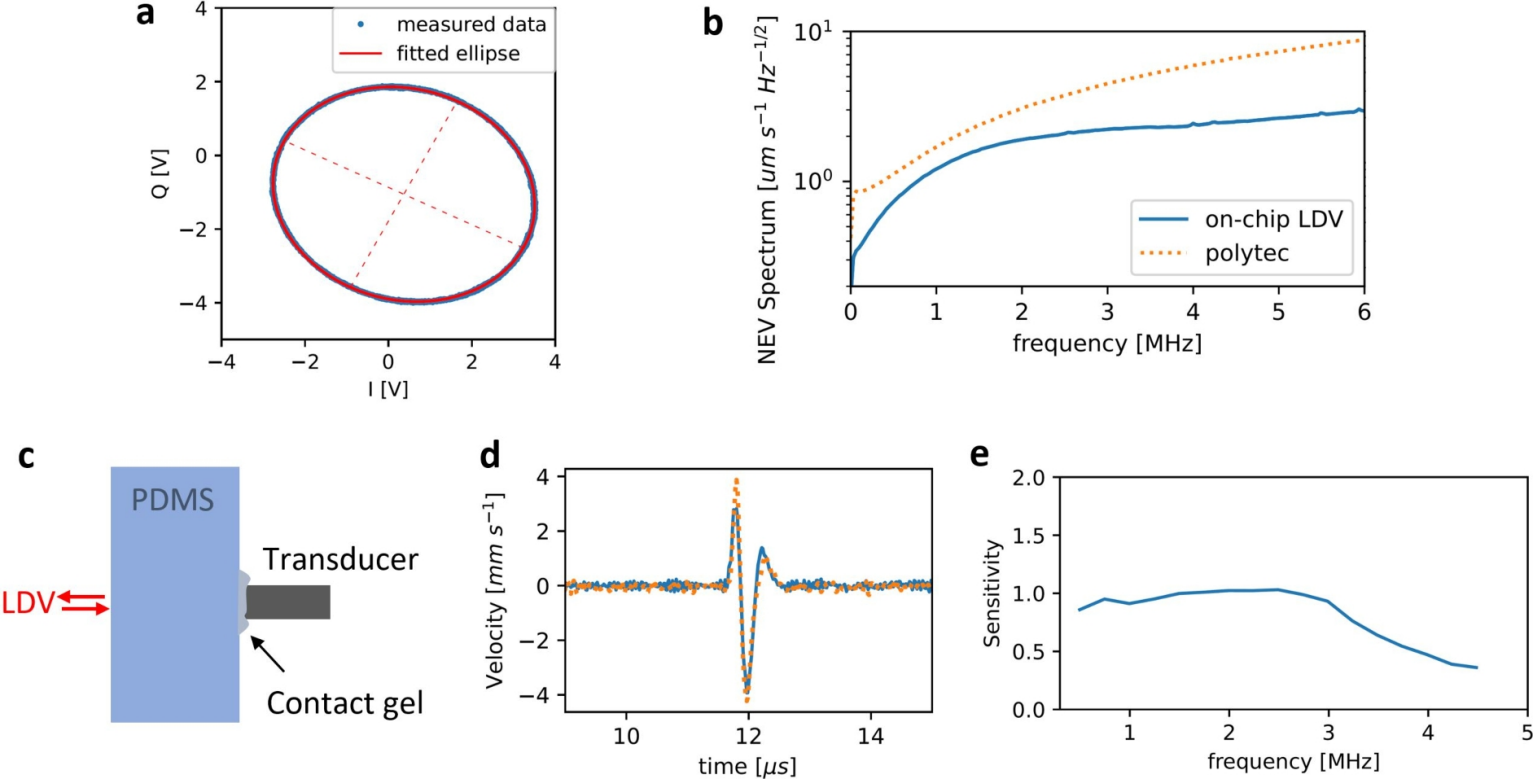
(**a**) Reference measurement of the I and Q signals and fitting of the ellipse before demodulation. (**b**) Measured velocity noise floor for the polytec and the on-chip LDV after demodulation. (**c**) Schematic of setup used to compare pulse responses. The LDV is swapped between the Polytec and the on-chip LDV. (**d**) A pulse time trace measured by the Polytec (orange) and on-chip LDV (blue). (**e**) The sensitivity from the LDV compared to the polytec resulting from the spectrum comparison of the pulse response.

Note that for a perfect system, the I and Q points constitute a perfect circle when inducing vibration amplitudes larger than the wavelength (> 1550 nm). However, due to fabrication imperfections, DC offsets, and an elliptical shape are observed, as shown in Fig. 2a. These distortions can be compensated for as discussed in the section ’Photoacoustic Signal Processing and Imaging’ or through other methods^24^.

When an ultrasound wave impinges on the boundary of a sample, there is a small displacement of this surface. When this surface is probed by the measurement beam of the LDV, this displacement, *d*(*t*), causes a small path length change. From monitoring the phase change $\Delta \theta '(t)$ obtained after demodulation, the displacement can easily be calculated, with $\lambda = 1550~nm$, the wavelength of the probe beam.

6 $$\begin{aligned} \Delta d(t) = \frac{\lambda \Delta \theta '(t)}{4\pi } \end{aligned}$$

#### Performance characterization methods

To characterize the noise floor, the Noise Equivalent Velocity (NEV) spectrum (Fig. 2b) was estimated by processing a 1-second LDV-recording on a still silicone sample using the Welch method^25^. This analysis was performed for both a commercial LDV (Polytec OFV-534) and the on-chip LDV. To characterize the sensitivity spectrum and bandwidth of the chip-based LDV, a setup, as depicted in Fig.2c, was constructed to generate ultrasound pulses in the silicone. An ultrasound transducer was placed against a 12 mm thick silicone sample, using contact gel to ensure good ultrasound transmission. The transducer was driven using 50 ns, 10 V pulses at a repetition rate of 1 kHz, and time traces were recorded and averaged for both the polytec and chip-based LDV. After averaging for 100 seconds, the recorded time traces can be compared to give the sensitivity in Fig. 2d. Consider P(f) and C(f) to be the calculated FFT spectra of the time traces recorded by the Polytec and the chip-based LDV respectively. Knowing that the commercial LDV presents an almost flat unity sensitivity for ultrasound frequencies up to 10 MHz, the sensitivity of the chip-based LDV can be estimated as $S(f)=C(f)/P(f)$, as long as signals are present above the noise floor, (in this case up to 4.5 MHz).

### Pulsed laser diode

Conventional photoacoustic systems often use bulky and expensive pulsed laser sources to generate high-power nanosecond pulses. However, to enable compact photoacoustic imaging, we use a pulsed laser diode as a small and cheaper alternative. A commercially available pulsed laser driver (Picolas LDP-V 240-100) drives the pulsed laser diode (Osram, SPL S4L90A) with a wavelength of around 905 nm, generating optical pulses with durations between 100 and 500 ns and a peak optical power of 500 W, resulting in pulse energies ranging between 50 and 250 $\mu J$. Although the pulse energy of the pulsed laser diode is around one or two orders of magnitude lower compared to more expensive pulsed laser systems like laser diode-pumped optical parametric oscillators (OPO), a pulsed laser diode allows for higher repetition rates, which enables collecting a higher number of segments for averaging, thereby partially compensating for the lower signal^20^. In this paper, the pulsed laser is operated at a repetition rate of 1 kHz and a pulse width of around 400 ns resulting in approximately 0.2 W average power and around 200 $\mu J$of pulse energy, which is within the ANSI skin exposure safety limits^22^.

### Silicone sample

Photoacoustic signals are generated through the sudden absorption of optical energy in a sample, causing local heating and expansion leading to the emission of a propagating pressure wave. In this paper, a silicone sample was developed featuring a channel filled with an absorbing solution. By putting an ink-water solution in this channel, the 905 nm light is absorbed there and generates photoacoustic signals upon pulsed laser illumination. The silicone samples were made by pouring Polydimethylsiloxane (PDMS, Sylgard 184) in molds with a thickness = 10-13 mm, each containing rods with a diameter of 2 mm. After 12 hours of curing at 40 degrees Celcius, these rods were removed, resulting in an empty channel. The PDMS has a typical speed of sound of 1020 m/s^10,26^

It is important to note that the base of the mold was a glass surface to enable a smooth and specular reflective surface to enable efficient LDV detection. Various concentrations of water-based ink solutions (0.01 % - 1 % Black India ink) were employed in the channel to act as the absorber and thus the origin of the photoacoustic signals. The absorption of the 0.1 % ink solution was measured to have an extinction coefficient of 12.5 $cm^{-1}$.

### Photoacoustic signal processing and imaging

Due to imperfections in the photonic circuit and electrical amplification (resulting from an imbalance in the hybrid/photodetector or electrical amplifier circuit), the detected IQ points did not constitute a circle but rather an ellipse, with a DC offset. To enable accurate demodulation, a reference measurement of a large vibration was recorded with the LDV before each measurement. This allows the ellipse to be fitted, which can then be used to project all data points onto the unit circle before arctan demodulation, as seen in Eq. 5, ensuring accurate demodulation. An example of a recorded and fitted ellipse can be seen in figure 2a.

The sample and pulsed laser diode were attached to a scanning stage. Scanning the LDV beam in a line over the surface of the sample allows for a 2D photoacoustic reconstruction of the deposited energy by the excitation laser. A scanning pitch of 125 $\mu m$ was used and the demodulated signals were averaged over 1 second for each location and low-pass filtered with a cutoff frequency of 5 MHz to limit the noise. As stated in Eq. 6, the displacement can be calculated using the demodulated signal. Then, by taking the temporal derivative, an estimate of the velocity *u* of the sample’s surface can be retrieved. The pressure *p* measured at the surface can be estimated using the following relation with the acoustic impedance of the sample $Z_s>>Z_{air}$^27^:

7 $$\begin{aligned} p = \frac{Z_s}{2} u \end{aligned}$$

To reconstruct the image, we used a 2D time-reversal algorithm, implemented with the MATLAB package k-wave^28^. The algorithm uses the measured forward-propagation field to generate back-propagation fields, after which a Hilbert transform is applied to reconstruct initial pressures at time = 0. For the image reconstruction in this paper, we used a reported speed of sound in PDMS samples of 1020 m/s^10,26^. This speed of sound is confirmed when looking at the arrival time of the ultrasound pulse through a 12 mm thick sample (Fig. 2d, as recorded with the setup in Fig. 2c.)

## Results and discussion

### Silicon photonics-based LDV performance

Considering the noise floor expressed in Fig. 2b, it is clear that the on-chip LDV presents a noise floor on par with or even better compared to a commercial LDV when measuring on flat specularly reflective surfaces. This could be attributed to the optimization of the chip-based LDVs electronics for high collection efficiency, thereby minimizing electronic noise contributions. In contrast, commercial LDVs are typically designed to operate across a wide range of collection efficiencies, which may impact the noise performance for relatively high collection efficiencies.

Increasing the on-chip LDV’s output power towards the laser class 1 limit of 10 mW could further lower the detection limit. However, increasing the power in silicon photonics is limited by strong nonlinear effects, such as two-photon absorption, which increases the loss of high optical powers. Despite this, an output power of 10 mW is feasible for high optical input power and limited waveguide lengths. In the current system, however, saturation occurs during the electrical amplification for LDV output beam powers higher than 0.5 mW.

Using Eq. 7 the noise floor can be expressed as a noise equivalent pressure spectrum. It is, however, important to consider that the on-chip LDV has a limited bandwidth, due to the limited bandwidth of the amplifier electronics. The bandwidth can be seen in the measured sensitivity Fig. 2e. These results show that the sensitivity remains relatively constant (near unity) up to around 3 MHz, after which the sensitivity drops, resulting in a 3 dB bandwidth of around 3.5 MHz.

Considering a digital 5MHz low-pass filter, integration of the on-chip curve in Fig. 2b results in an estimated total NEV of around 4 mm/s and thus estimated total NEP of around 2 kPa using Eq. 7. Although the LDV performance is similar to commercial systems^16^, averaging is required to detect photoacoustic signals excited by the compact pulsed laser diode. Averaging will reduce the noise floor by a factor equal to the square root of the number of accumulations, but increases the accumulation time linearly. This can impact performance due to motion artifacts, especially for in vivo samples.

In vivo samples, however, generally do not have specular reflective surfaces and often present rough, scattering surfaces, degrading the collection efficiency and thus the performance of LDV systems. Since in vivo ultrasound detection with a commercial 1550 nm LDV has been demonstrated^29^, it indicates that an improved optical system design and packaging could allow for in vivo detection of ultrasounds^30^. A recent demonstration shows measurement on bare skin using an optical system with an increased numerical aperture (NA)^24^. High NA optics, however, have a smaller depth of focus, making it difficult for in vivo detection, which may require automatic focusing feedback loops. Another approach, often used in various LDV-based photoacoustic demonstrations, is using a sample preparation method to enhance reflection, such as applying oil or water^31,32^.

### Non-contact photoacoustic imaging with silicon photonics-based LDV

**Fig. 3:**
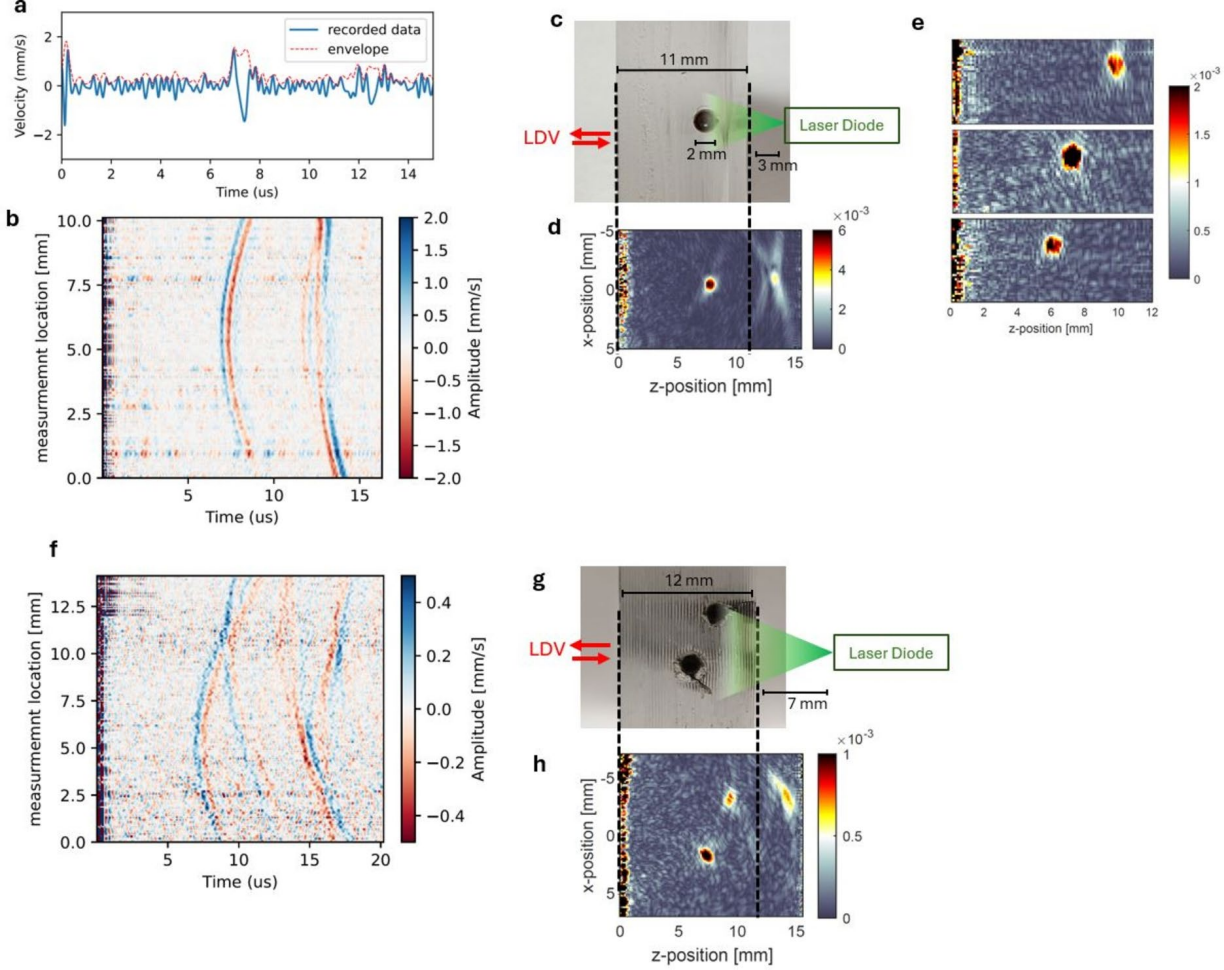
(**a**) Time trace of a recorded and demodulated photoacoustic signal and its envelope calculated by the Hilbert transform. (**b**) Plot of the photoacoustic signal for different locations along the scanning direction of the probe beam on the surface of the sample depicted in (**c**). (**d**) Time reversal reconstruction of (**c**) using the data of (**b**). (**e**) Reconstructed images for channels with their centers at different depths of 5.8, 7 and 9.6 mm. (**f**) Scanning data for a dual channel sample (depicted in **g**). (**h**) Time reversal reconstruction for the dual channel sample. All of the channels in this figure are 2 mm in diameter.

Using the setup, described in the Methods section and depicted in Fig. 1a, photoacoustic signals can be recorded. Fig.3a shows a time trace of a demodulated and averaged photoacoustic signal recorded by the on-chip LDV on a single-channel sample, with the channel containing a 1 % ink-water solution and averaging over 1 second after demodulation. The envelope is calculated using the Hilbert transform and is used as input to the reconstruction algorithm. Time zero indicates the firing of the pulse and shows an interference with the detection system. After a period of around 7 $\mu s$, a small movement of the surface is detected by the LDV. The delay between excitation and signal arrival indicates the distance between the signal origin and the detection location.

In order to create a 2D photoacoustic image, the LDV beam was scanned along a line on the surface of the sample. Figure 3b shows the detected photoacoustic signals at different locations for an embedded light-absorbing channel (depicted in figure 3c). With a scanning pitch of $125~\mu m$, below the cutoff acoustical wavelength of our system ($\lambda _{@5~MHz} \approx 200~\mu m$), and a total distance of around 1 *cm*, signals were recorded at 80 different locations. From figure 3b, it can be seen that for some locations, the signal-to-noise ratio is better than others. This is due to variations in the collection of optical power after reflection from the sample surface, often caused by impurities or dust on the detection surface.

Figure 3b shows a clear relation between the measurement location and signal delay, but also a secondary signal originating from the reflection of the generated acoustic signal from the backside of the sample.

Using the time reversal algorithm mentioned in the Methods section, a reconstruction image can be made (Fig. 3d). This image shows the correct location of the embedded channel relative to the detection locations and includes a mirror image due to the reflection from the backside of the sample. Reconstruction images at different depths (Fig. 3e) demonstrate the capability to determine the position and depth of the channel. It can, however, be noted that the amplitude differs between these locations. This is due to the influence of realigning the excitation source and LDV for different samples and the varying optical fluence and acoustic divergence at different depths.

Fig. 3f and h show the detected signals and reconstruction for a sample with two embedded channels (Fig. 3g). To illuminate both channels, the laser diode was placed further away from the sample, resulting in weaker illumination and thus lower signal generation. The illumination profile of the pulsed laser diode determines the field of view (FOV) along with the measurement locations of the LDV. The beam divergence (full-width half maximum) of the pulsed laser diode is 25° perpendicular to the ink channel and 10° parallel to the ink channel, such that there is a larger FOV in the scanning direction of the LDV. However, it can be noted that out-of plane excitation is present and could reduce performance for photoacoustic imaging, but here remains limited due to the small divergence angle.

Due to the large influence of the illumination profile of the excitation source, a scaled system requires the design of a uniform illumination profile along the LDV detection points. This can be achieved by incorporating multiple excitation sources or advanced optics. While in this paper excitation and detection occur at opposite sides of the sample, many applications require same-side excitation and detection. In this scenario, the interference between the excitation and detection systems could affect the detection of shallow absorbers and thus should be mitigated. However, it is expected that the interference will already be lower for same-side excitation since the excitation light is not sent directly into the LDV system. The remaining influence can be further reduced by filtering of the light, which is possible due to the difference in detection and excitation wavelengths^32^. Same-side detection introduces additional challenges for contact-based detectors, as their contact nature often limits the efficient delivery of excitation light beneath them. However, this limitation does not apply when using optical detection methods. With optical detection, the detection locations can overlap with the excitation illumination, for example by using dichroic mirrors. This overlap allows for more flexible and efficient setups, improving the overall performance of the system. However, it is important to note that the imaging depth differs for same-side excitation due to the equal optical and acoustic propagation lengths, in contrast to opposite site excitation.

Considering a bandwidth of 3.5 MHz of the LDV-based detection system and assuming an impedance of 1.02 MRayl, the theoretically estimated resolution of the photoacoustic images is limited to $0.8\lambda _{c}\approx 230~\mu m$, with $\lambda _{c}$being the acoustic wavelength for the cutoff frequency^33,34^.

**Fig. 4:**
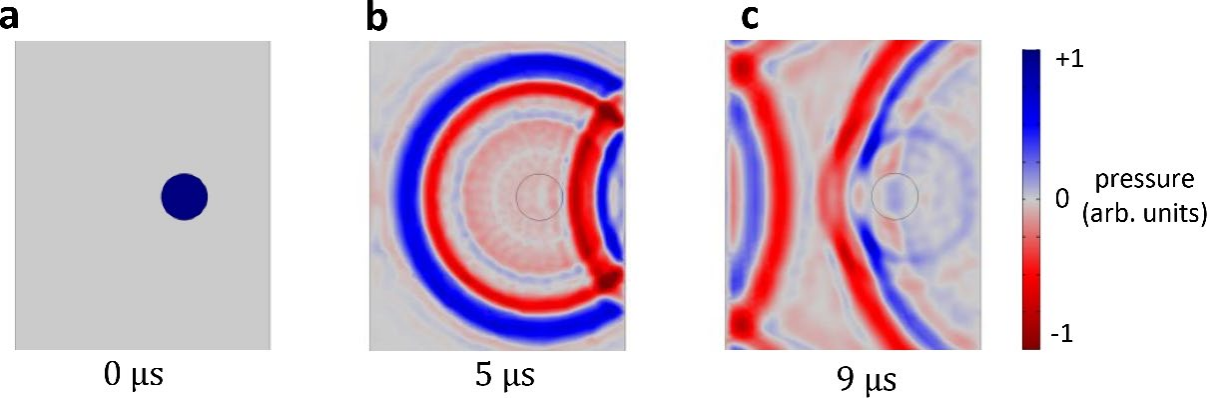
2D COMSOL simulation of acoustic propagation in a geometry similar to Fig. 1 e for an initial pressure distribution with increased pressure inside the tube filled with a water-based solution surrounded by PDMS at (**a**) $t=0~\mu s$, (**b**) $t=5~\mu s$ and (**c**) $t=9~\mu s$.

In Fig. 3b, it can also be noted that the reflected signal has a different shape compared to the primary signal. This secondary signal shows some ’splitting’ of the acoustic pulse into two contributions arriving one after the other for the measurements locations near the location of the channel. A simulation using COMSOL of the scalar wave equation for a simplified acoustic propagation problem reveals that the splitting originates from the transmission of the reflected signal through the channel (Fig. 4). The simulation assumes a uniform acoustic impedance of the PDMS of around 1.02 MRayl^10,26^, an acoustic impedance of the channel of 1.5 MRayl, and a uniformly increased initial pressure across the channel (Fig. 4a). The geometry is similar to the dimensions in Fig. 3c and soft sound boundary conditions are assumed for the PDMS/air interface. After propagation for around $5~\mu s$, Fig. 4b shows that the primary signal is propagating and almost reaching the detection surface (left), while the secondary signal has appeared due to the reflection from the backside. Note that after $5~\mu s$, no splitting of the reflected signal can be observed. However, the shape of the reflected signal after it has passed by the channel, as can be seen in Fig. 4c, shows the splitting of the reflected signal. The first contribution of this reflected wave is the signal passing through the channel with a higher speed of sound while the second contribution is the diffracted signal going ’around’ the channel.

**Fig. 5:**
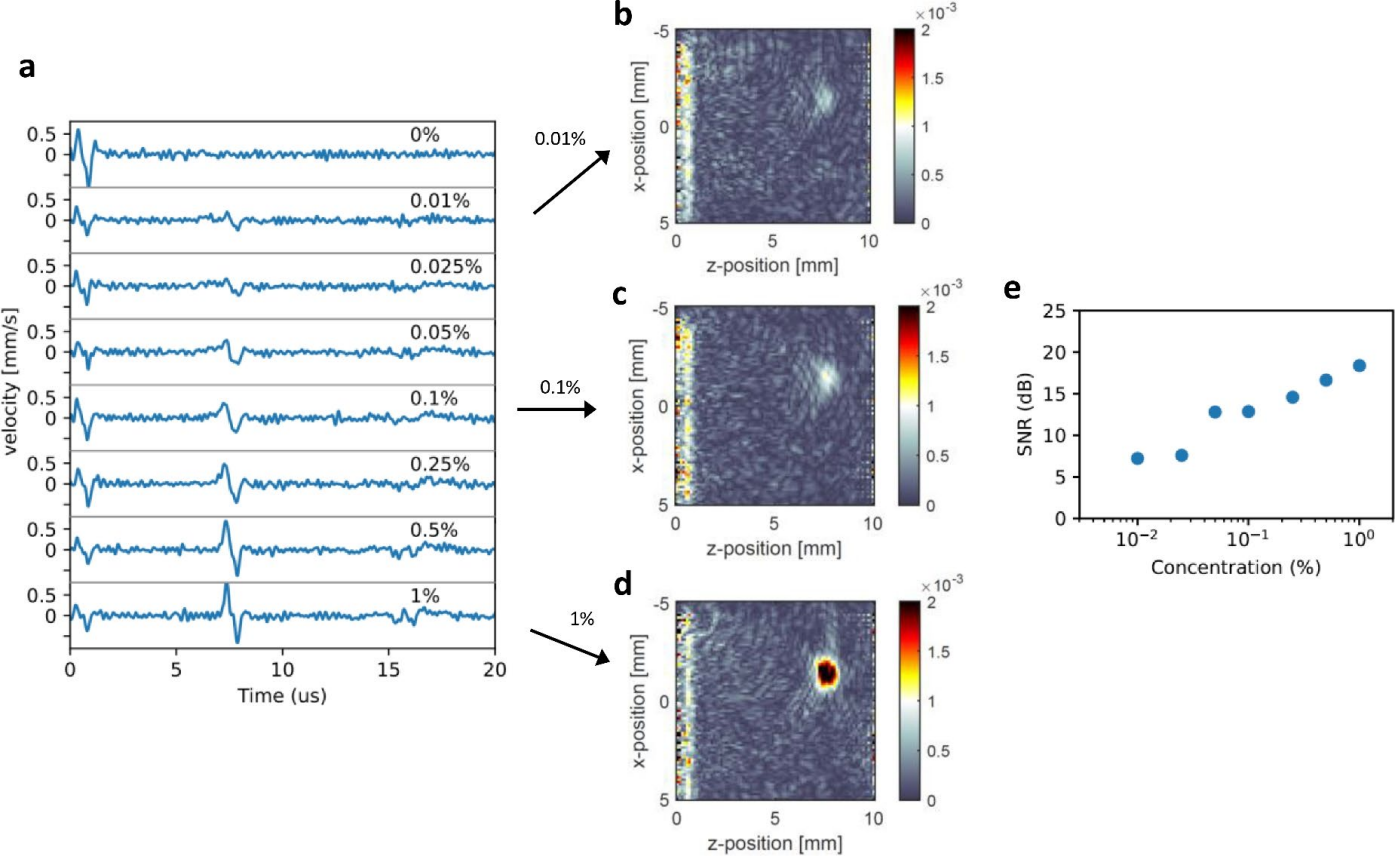
(**a**) Signal time traces of the recorded velocity of the surface after photoacoustic excitation for different ink concentrations inside the sample. Increasing the concentrations results in stronger signals. The 0.1 % ink solution was measured to have an absorption of 12.5 $cm^{-1}$ at a 905 nm wavelength. (**b**, **c**, **d**) Show image reconstructions for different ink concentrations showing reduced contrast for lower concentrations. (**e**) SNR for the different concentrations.

While the results from Fig. 3 were obtained using a 1 % ink concentration, resulting in an absorption coefficient well above the physiological range, Fig.  5a demonstrates the dependence of the amplitude of the photoacoustic signals on the absorption of the ink solution. These results were obtained by aligning the sample and the LDV at the beginning of the measurement. After the initial alignment, we only changed the ink concentration in the setup to observe its effects on the signal strengths. Fig. 5e shows that the signal-to-noise ratio (SNR) clearly increases with higher ink concentration, which is also visually evident from the reconstructed images in Fig.5b. The SNR is defined by taking the maximum velocity of the pulse after the Hilbert transform divided by the RMSE-error. We detect small photoacoustic signals for ink concentrations down to 0.01 % corresponding to an absorption coefficient of around 1.2 $cm^{-1}$. Unlike the PDMS sample, in vivo samples generally exhibit optical scattering and absorption, which affect the illumination and, consequently, the imaging strength and depth. Although not verified experimentally, it is estimated that in vivo absorbers could be detected up to a depth of a couple of millimeters deep with an LDV considering good reflected power^35^. Since the measured signals for absorption coefficients within the physiological range at 905 nm ($2-10~cm^{-1}$)^2^. show only a limited strength, a higher excitation power may be necessary for in vivo demonstrations.

## Conclusion and outlook

In this paper, a photonics-enabled homodyne LDV was developed for compact and contactless ultrasound detection. We demonstrated a lab setup for contactless photoacoustic imaging by scanning the probe beam over a flat sample’s surface while using a small and inexpensive pulsed laser diode as the excitation source. Advanced packaging methods can further enhance the total compactness of the device, as demonstrated in^24^, where a micro-optical bench includes a laser source, an optical isolator, and micro ball lenses to compactly co-package the laser source with the LDV.

The samples used in this paper were clear, flat PDMS-based samples with an ink solution-filled channel embedded, acting as the absorber. For these samples, the photonics-based detection system presented a detection limit on par with or even better than commercial systems, with a bandwidth of up to around 3.5 MHz. Moving to in vivo experiments, where the sample surfaces usually present roughness and anisotropically reflect light, requires addressing the reduced collection efficiency caused by surface scattering and roughness. This can be achieved by either enhancing the optical design efficiency of the LDV or by enhancing reflection by applying water or oil to planarize the sample’s surface.

Although the demonstration in this paper only uses a single on-chip LDV beam, requiring scanning along the surface to enable photoacoustic imaging, photonic integration holds potential for scaling to multi-beam LDV layouts, with element pitches down to a couple of $\mu m$^36^, which could eliminate the need for scanning. It is important to note that while scaling the number of LDV detection points to a few beams has been demonstrated^18^, achieving hundreds of elements, as seen in commercial contact-based ultrasound probes, remains challenging due to the large number of electrical connections required. Recent developments suggest reducing the number of connections through on-chip synthetic array heterodyning^37^ or focusing on advanced packaging for managing a large number of connections.

Our demonstration allowed for varying the absorber concentrations, showing relatively small signal strengths for physiological absorption coefficients in a non-scattering and non-absorbing medium. This suggests that for in vivo samples, imaging depth will be limited due to additional scattering and absorption effects. However, this depth could be improved by optimizing the overall excitation power while staying below safety limits. This optimization could involve combining multiple small laser diodes or using high-power pulsed lasers. Additionally, the illumination profile, which determines the system’s FOV, must be considered to enhance imaging performance.

For biomedical applications where blood acts as a contrast agent, future implementations could include multi-wavelength excitation which could yield quantitative information on the local oxygenation^3^.

In summary, this work evaluates the performance of a silicon photonics-based LDV for ultrasound detection within the context of photoacoustic imaging. It also includes a lab-based experiment demonstrating the potential of silicon photonics-enabled LDVs for compact and contactless ultrasound detection in photoacoustic imaging.

## Data Availability

The dataset and signal processing algorithms are available on github.com/edieussa/PA_Data_and_Figures or will be available from the corresponding author upon request.
